# Renninger’s Gedankenexperiment, the collapse of the wave function in a rigid quantum metamaterial and the reality of the quantum state vector

**DOI:** 10.1038/s41598-018-27759-6

**Published:** 2018-06-25

**Authors:** Sergey E. Savel’ev, Alexandre M. Zagoskin

**Affiliations:** 1 Department of Physics, Loughborough University, Loughborough, LE11 3TU United Kingdom; 2 Theoretical Physics and Quantum Technologies Department, Moscow Institute for Steel and Alloys, 119049 Moscow, Russia

## Abstract

A popular interpretation of the “collapse” of the wave function is as being the result of a local interaction (“measurement”) of the quantum system with a macroscopic system (“detector”), with the ensuing loss of phase coherence between macroscopically distinct components of its quantum state vector. Nevetheless as early as in 1953 Renninger suggested a Gedankenexperiment, in which the collapse is triggered by *non-*observation of one of two mutually exclusive outcomes of the measurement, i.e., in the absence of interaction of the quantum system with the detector. This provided a powerful argument in favour of “physical reality” of (nonlocal) quantum state vector. In this paper we consider a possible version of Renninger’s experiment using the light propagation through a birefringent quantum metamaterial. Its realization would provide a clear visualization of a wave function collapse produced by a “non-measurement”, and make the concept of a physically real quantum state vector more acceptable.

## Introduction

The central element of the transition from quantum to classical behaviour (e.g., during the measurement) is the loss of phase coherence between the components of the wave function corresponding to macroscopically distinguishable states of the system^1^. The loss is popularly ascribed to the interaction of the quantum system with a macroscopic environment. The details of this interaction were initially considered irrelevant as reflected in the von Neumann reduction postulate^2^, which describes strong projective measurements. Detailed attention to the details of this process was attracted by later developments of experimental techniques and theory (quantum non-demolition measurements; continuous weak measurements^3^; observation of quantum coherent effects in artificial structures on at least mesoscopic scale^4^; for review see, e.g.^3,5,6^). In certain approaches the decoherence is responsible for the selection of quantum observables, which have macroscopic counterparts (“quantum Darwinism”)^7^.

**Figure 1:**
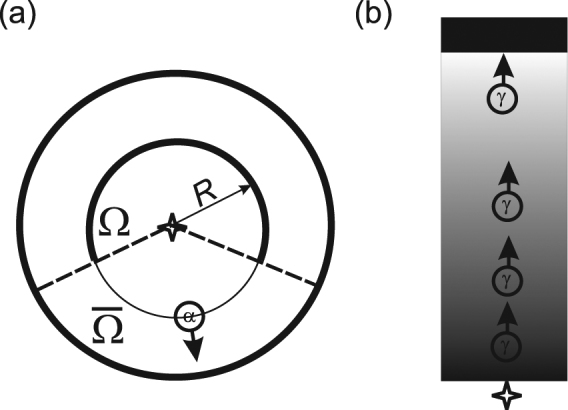
(**a**) The version of Renninger’s Gedankenexperiment^8^ proposed by Cramer^10^. The source of $\alpha$-particles with speed $v$ (centre) is surrounded by two scintillating screens. The absence of a flash on the inner screen at the moment $t=R/v$ after emission indicates the collapse of the initially spherically symmetric wave function of the emitted particle to the complement $\bar{{\rm{\Omega }}}$ of the solid angle ${\rm{\Omega }}$ covered by the inner screen. (**b**) Photons emitted by a single-photon source at the bottom of a quantum metamaterial slab travel towards the detector (black bar) at its top.

Nevertheless a remarkable, but often overlooked, “negative-result Gedankenexperiment” proposed by Renninger in 1953^8,9^ underlines that actually the collapse should follow also in case of a *non-*detection, and therefore no interaction with a detector. A simple version of his argument is given by Cramer^10^. There a radioactive atom at the origin, surrounded by a spherical scintillator screen, inside which is placed a screen of a smaller radius $R$, covering a solid angle ${\rm{\Omega }} < 4\pi$ (Fig. 1(a)). After the decay the emitted particle (e.g., an $\alpha$ particle) has a definite speed $v$ and equal probability to go in any direction and must be described by a spherical wave. But if at the moment $t=R/v$ there is no flash on the smaller screen, this means that the particle was emitted into the complement $\bar{{\rm{\Omega }}}$ of the angle ${\rm{\Omega }}$, and its wave function must collapse to $\bar{{\rm{\Omega }}}$ - *even though no interaction with a macrosopic detector took place as yet*.

On the other hand, Dicke^11^, considering a different layout - detection or non-detection of a photon scattered by an atom in a Heisenberg microscope-type setup,- showed that a *virtual* process of photon interaction with the atom could lead to the change of atom energy dependent on whether the photon was eventually absorbed by a given detector, and thus related the state collapse to this interaction. In a similar way, an off-resonance photon interacting with an array of quantum bits is predicted to produce identical phase shifts in their states, which affects the collective variable of the array (e.g., its total magnetic moment) and can be read out even if the photon is not absorbed^12^, providing another example of a quantum non-demolition measurement.

As pointed out by Renninger, these results are more naturally understood if the wave functions (or quantum state vectors) are considered as objectively existing, nonlocal physical objects subject to nonlocal interactions, rather than a measure of somebody’s knowledge about the status of a microscopic system expressible in macroscopic terms. The latter (“epistemic” or “positivist”^2^) interpretation was to a certain extent undermined after 2012 (“no-go” theorems^13–16^ and related experiments^17^), though it still cannot be excluded, since all interpretations consistent with the equations of quantum mechanics provide the same predictions, and the choice between them is more the question of what to consider “objective” or “intuitively clear”. Nevertheless the search for the least contrived interpretation, and its best experimental visualization, is justified - from the point of view of clarity as much as from the hope that such a search may discover deviations from quantum theory and thus lead to observable differences between interpretations.

In this paper we propose a version of Renninger’s experiment, which uses a quantum metamaterial (QMM) - a macroscopic, quantum coherent optical medium - and show that the observable statistics of photons passing through such a medium presents a clear visualization of this effect, which supports Renninger’s point of view that a quantum state vector is an “element of reality” possessing both wavelike and particlelike qualities simultaneously. A QMM is an artificial medium, the optical properties of which depend on its local quantum state, and which maintains its global quantum coherence long enough for an electromagnetic pulse to travel across it^18^. First experimental prototypes of such media based on superconducting qubits and operating in the microwave range have been built^19,20^.

## Model

For our current purpose the actual implementation of a QMM is irrelevant. We thus consider a QMM slab which has two quantum states, $\mathrm{|1}\rangle$ and $\mathrm{|2}\rangle$, characterized by different refractive indexes ${n}_{1}$ and ${n}_{2}$ respectively. In other words, a photon propagates through the system either with the speed ${c}_{1}=c/\sqrt{{n}_{1}}$ or ${c}_{2}=c/\sqrt{{n}_{2}} < {c}_{1}$, depending on the system’s quantum state. Note that we do not assume that $\mathrm{|1}\rangle$ and $\mathrm{|2}\rangle$ are the eigenstates of the Hamiltonian: on the contrary, quantum beats between them play an important role in the following (cf.^18^). An ideal source of single photons is set at the one end of the QMM slab, and an ideal detector at the other (Fig. 1(b)).

At the degeneracy point of states $\mathrm{|1}\rangle$ and $\mathrm{|2}\rangle$ the QMM state $|\psi \rangle ={a}_{1}\mathrm{|1}\rangle +{a}_{2}\mathrm{|2}\rangle$ is described by the Schrödinger equation

1 $$i\hslash \partial |\psi \rangle /\partial t=\hat{H}|\psi \rangle $$

with the Hamiltonian

2 $$\hat{H}=\hslash \omega \mathrm{(|1}\rangle \langle \mathrm{2|}+\mathrm{|2}\rangle \langle \mathrm{1|)}$$

This equation is readily solved resulting in

3 $$|\psi \rangle =\,\cos (\omega t+{\varphi }\mathrm{)|1}\rangle -i\,\sin (\omega t+{\varphi }\mathrm{)|2}\rangle \mathrm{.}$$

**Figure 2:**
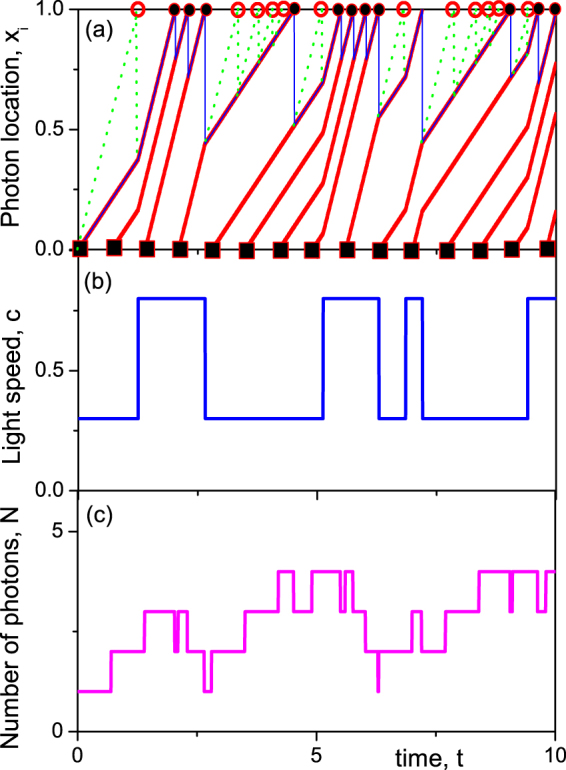
(**a**) A realization of photon trajectories shown in red simulated for ${c}_{1}=0.8$, ${c}_{2}=0.3$, $\omega =0.7$, $\tau =0.3$. The squares at the bottom of the plot represent the events $(x=\mathrm{0,}\,t=m\tau )$ of photon injections while filled (open) circles at the top of the slab represent successful (unsuccessful) measurements of a photon. The green dotted lines show the unrealized trajectories which would occur if the nearest-to-top photon had travelled with speed ${c}_{1}$. (**b,c**) A particular realization of photon velocity switches and photon number in the slab corresponding to trajectories shown in (a).

Now let photons be emitted one by one at times $t=\mathrm{0,}\tau ,\ldots m\tau ,\ldots$ (Fig. 2(a), filled squares) and detected after their passage through the QMM. The quantum state of the latter is set to $\mathrm{|1}\rangle$ (i.e., ${\varphi }\,=\,0$) at $t\,=\,0$. The first measurement occurs at time ${t}_{1}=L/{c}_{1}$ (marked by the first open circle at the slab top surface, Fig. 2(a)) and if a photon were detected (output 1, which is not the case for the particular simulation shown in Fig. 2(a)), the wave function $|\psi \rangle ({t}_{1})$ would have collapsed to the state $\mathrm{|1}\rangle$ (producing ${\varphi }({t}_{1})=-\,\omega {t}_{1}$). In the case shown in Fig. 2(a) the photon was not detected at $t={t}_{1}$ (output 2). Therefore the QMM state collapsed to $\mathrm{|2}\rangle$ (i.e., ${\varphi }({t}_{1})=-\,\omega {t}_{1}+\pi \mathrm{/2}$), and the photons’ speed $c\mathrm{(0} < t < {t}_{1})$ was set at ${c}_{2}$. Therefore the measured output 2 sets the speed of photons $c={c}_{2}$ retrospectively, for the times $0 < t < {t}_{1}$, i.e., *before* to the measurement event.

The position ${x}_{{\rm{front}}}$ of the nearest-to-surface photon (the front of photon distribution, blue line in Fig. 2(a)) at ${t}_{1}$ would be ${x}_{{\rm{front}}}={c}_{1}({t}_{1}-\tau )$ (measurement outcome 1: ${x}_{{\rm{front}}}$ would be the location of the second photon injected in the slab at $t=\tau$, since the first photon would had been absorbed by the detector) or ${x}_{{\rm{front}}}={c}_{2}{t}_{1}$ (since the measurement shows no photon detection at $t={t}_{1}$, the measurement outcome 2: ${x}_{{\rm{front}}}$ would be the position of the first photon propagating with speed ${c}_{2}$). Therefore, we can fix the time of the next expected measurement ${t}_{2}={t}_{1}+(L-{x}_{{\rm{front}}})/{c}_{1}$. It can either detect a photon (output 1) or not (output 2). If photon is detected, then the QMM state becomes $|\psi \rangle ({t}_{2})=\mathrm{|1}\rangle$, retrospectively setting $c({t}_{1} < t < {t}_{2})={c}_{1}$. Otherwise the state collapses to $|\psi \rangle ({t}_{2})=\mathrm{|2}\rangle$ and $c({t}_{1} < t < {t}_{2})={c}_{2}$. Continuing the process leads to a series of switches between states with $c={c}_{1}$ and $c={c}_{2}$, an instance of which is shown in Fig. 2.

In order to complete the description of simulations, we have to describe the procedure of measurements, which collapse the wave function. For example, at the moment $t={t}_{1}-0$ (i.e., just before the collapse) we have $|\psi \rangle =\,\cos (\omega {t}_{1}\mathrm{)|1}\rangle -i\,\sin (\omega {t}_{1}\mathrm{)|2}\rangle$. Therefore, the probability to collapse to the state $\mathrm{|1}\rangle$ is ${P}_{1}={\cos }^{2}(\omega {t}_{1})$, while that of collapsing to the state $\mathrm{|2}\rangle$ is ${P}_{1}={\sin }^{2}(\omega {t}_{1})$. A random number $p$ within the unit interval $0 < p < 1$ was generated and compared with ${P}_{1}$. If $p\, < \,{P}_{1}$, the system was set to state $\mathrm{|1}\rangle$, otherwise to state $\mathrm{|2}\rangle$. For the realization shown in Fig. 2(a) the random number was $p > {\cos }^{2}(\omega {t}_{1})$, therefore, the system collapsed to state $\mathrm{|2}\rangle$, which defines the speed of light $c={c}_{2}$ before the measurement. In addition, the measurement result sets the wave function after the measurement, which, in our particular case, is set to $|\psi \rangle =\,\sin (\omega (t-{t}_{1}\mathrm{))|1}\rangle -i\,\cos (\omega (t-{t}_{1}\mathrm{))|2}\rangle$. At the time ${t}_{2}$ of the second measurement the wave function evolves to $|\psi \rangle =\,\sin (\omega ({t}_{2}-{t}_{1}\mathrm{))|1}\rangle -i\,\cos (\omega ({t}_{2}-{t}_{1}\mathrm{))|2}\rangle$, thus, the probability of the collapse to the state $\mathrm{|1}\rangle$ is ${P}_{1}={\sin }^{2}(\omega ({t}_{2}-{t}_{1}))$ at $t={t}_{2}$. So a new random number was generated and compared with ${P}_{1}$, resulting in either the collapse to the state $\mathrm{|1}\rangle$ (if $p < {P}_{1}$, which occurs for the realisation shown in Fig. 2a) or to the state $\mathrm{|2}\rangle$. This discrete stochastic process allows us to determine both the moments of expected photon arrival times and the outcomes of state collapse (or non-collapse) at these moments.

Red lines in Fig. 2(a) represent photon trajectories, with the dotted green line showing possible trajectories which would have been realized if the photon had been detected (the red open circle at the point $(L,{t}_{1})$). Open/filled circles at $x=L$ show negative/positive photon detection. Corresponding changes of the photons’ speed due to the shifts in the QMM refractive index are shown in Fig. 2(b). The fluctuations of photon number in the QMM caused by this are shown in Fig. 2(c).

## Results

**Figure 3:**
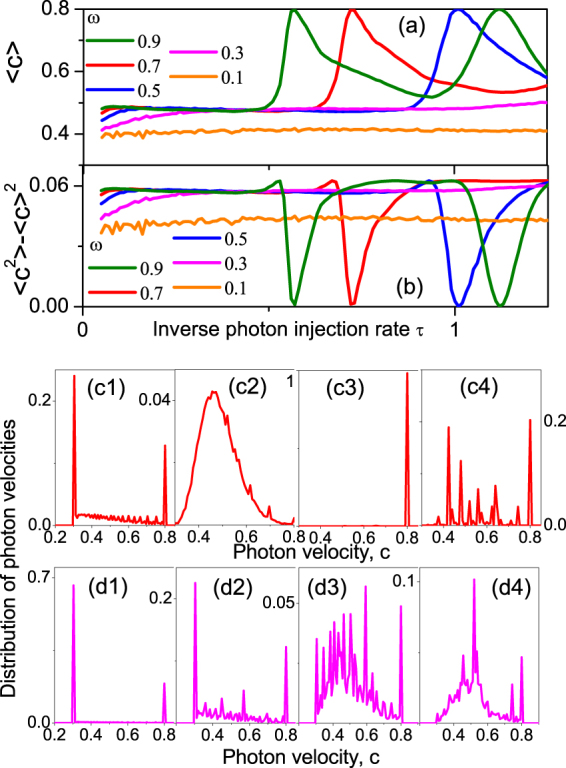
(**a**) The mean photon speed $\langle c\rangle$ averaged along *all* photon trajectories (during simulation time ${t}_{{\rm{\max }}}=50000$) and (**b**) its standard deviation $\langle {c}^{2}\rangle -{\langle c\rangle }^{2}$ both as a function of inverse photon injection rate $\tau$ for ${c}_{1}=0.8$, ${c}_{2}=0.3$ and $\omega$ shown in the legend. A very weak increase in $\langle c\rangle$ with $\tau$ at a slow quantum-slab dynamics ($\omega \lesssim 0.3$) is replaced by one or several peaks in $\langle c\rangle (\tau )$ and corresponding drops to zero in the standard deviation at a faster slab dynamics ($\omega \gtrsim 0.5$). Panels (c1-c4) show the distribution of the speed $\bar{c}$ averaged along *only one* photon trajectory (in contrast to (a) where averaging was done with respect to all photon trajectories) for ${c}_{1}=0.8$, ${c}_{2}=0.3$, $\omega =0.7$ as well as $\tau =0.05$ (c1), $\tau =0.3$ (c2), $\tau =0.72$ (c3) and $\tau =1.1$ (c4). A distribution with two clear maxima at $c={c}_{2}=0.3$ and $c={c}_{1}=0.8$ seen at high injection rates ($\tau =0.05$) transforms to a function with a smooth maximum located between ${c}_{1}$ and ${c}_{2}$ at intermediate injection rate $\tau =0.3$. Further increase of $\tau$ (panel c3) results in a distribution ${P}_{c}(\bar{c})$ with a very narrow maximum at $\tau$ when $\langle c\rangle (\tau =\mathrm{0.72)}={c}_{1}$ which is also consistent with the standard deviation $\langle {c}^{2}\rangle -{\langle c\rangle }^{2}$ dropping to zero in panel (b). When increasing $\tau$ further, a multiple-peak (“chaotic”) distribution of $\bar{c}$ develops (c4). Panels (d1-d4) show the same as (c1-c4) but for $\omega =0.3$ where there is no peak in $\langle c\rangle (\tau )$. An “ordered” two peak distribution of the single-trajectory-averaged photon speed $\bar{c}$ transforms to more and more “chaotic” multi-peak distributions with a background offset [see (d2-d4)] which is consistent with very weak dependence of $\langle c\rangle (\tau )$ and its standard deviation (see magenta curve in (b)).

**Figure 4:**
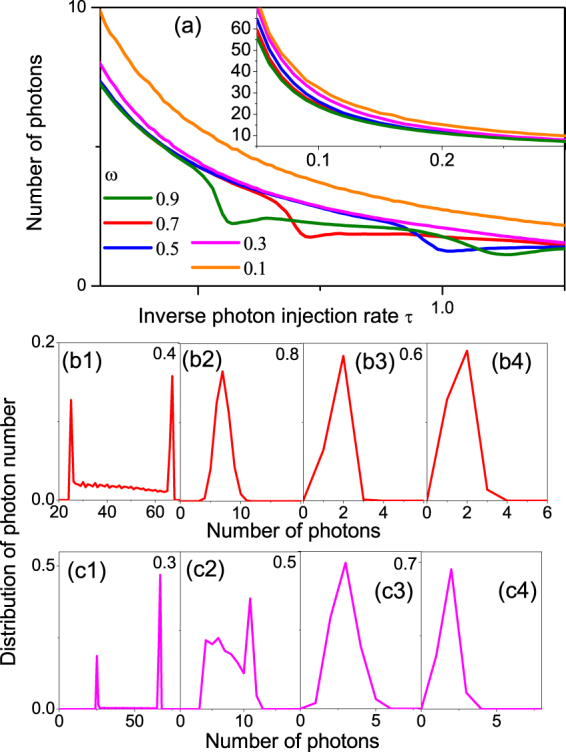
Mean photon number $\langle N\rangle$ (main pannel) in the quantum slab averaged over all simulation time (${t}_{{\rm{\max }}}=50000$) as a function of the inverse photon injection time $\tau$ and its standard deviation (inset in a) for the same parameters as in Fig. 2(a). Clear drops in photon numbers occur for the injection rates where $\langle c\rangle$ exhibits peaks. Panels (b1–b2) show a distribution of $N(t={t}_{M}=M{\rm{\Delta }}t)$ with ${\rm{\Delta }}t=0.002$ and $M$ from 1 to $2.5\,\times \,{10}^{7}$ (note, $\omega =0.7$ as in Fig. 2(c1–c4)). A two peak distribution for the high photon injection rate $\mathrm{1/}\tau$ transforms to a one-peak distribution with the peak location shifting to a low phonon number when $\tau$ increases. Panels (c1–c4) are the same as (b1–b4) but for $\omega =0.3$, i.e., correspond to (d1–d4) in Fig. 2. For $\tau =0.3$ and $\omega =0.3$ a more “chaotic” distribution occurs in (c2).

The fluctuations of the photon speed and phonon number in the QMM are caused by the interplay between state collapses by negative/positive photon detection events and the quantum beats between the QMM states with different refractive indices. Their statistics will therefore bear signatures of these events and can serve as a visualization of Renninger’s Gedankenexperiment. The results were obtained by numerical simulation and are presented in Figs 3, 4.

The average photon speed and its dispersion (Fig. 3(a,b)) strongly depend on the frequency of quantum beats in the QMM, $\omega$, versus the photon emission rate, $\mathrm{1/}\tau$. Looking at the time-averaged photon speed in the QMM (Fig. 2(a,b)), we see that at certain emission rates the average speed is fixed at the maximum, and its dispersion zero. This means that the QMM is also fixed in the state $\mathrm{|1}\rangle$ with the smaller refractive index, ${n}_{1}$, due to repeated measurements. Despite a superficial similarity to quantum Zeno effect^21^, this is the result of a resonance, occurring at $\omega \tau \approx 9.5\,m,m=\mathrm{1,}\,\mathrm{2,}\,\mathrm{...,}$ when the estimated “fast” detection times align with the quantum beats. (For “slow” detection times this would not work, since “fast” detections would happen first, - and it does not, since there is no fixing the system in the state $\mathrm{|2}\rangle$).

This view is confirmed by the analysis of the dependence of the distribution function of photon speed along a *single* photon trajectory on the photon emission time constant $\tau$ (Fig. 3(c,d)). For the fast photon emission rate (c1, d1) the average speed has two sharp peaks exactly at the limiting values, ${c}_{2}=0.3$ and ${c}_{1}=0.8$ for both relatively fast ($\omega =0.7$, panel (c1)) and slow ($\omega =0.3$, panel (d1)) quantum beats. *This* behaviour is indeed related to quantum Zeno effect. In that effect^21^ a frequently repeated strong measurement of certain quantum variable freezes the system in the eigenstate of this variable by repeated projections into this eigenstate. In our case, the preceding photons registered by the detector would repeatedly collapse the QMM state while the photon in question is traveling through it. Note that the weight of the “slow” state $\mathrm{|2}\rangle$ is the greater, and it grows as the photon emission (and therefore detection) rate increases compared to the quantum beat frequency: the Zeno effect tends to fix the system in the state with the *higher* refractive index (see also the trends of the curves in Fig. 3(a,b) as $\tau \to 0$). As the emission rate drops, this effect subsides, the single-trajectory velocity distribution broadens (panels (c2), (d2–3)). Then there may appear the resonant “anti-Zeno” effect (c3), fixing the QMM in the state with the *lower* refractive index, and eventually a chaotic distribution (c4,d3,d4).

The photon numbers in the QMM are similarly affected by the interplay between positive- and negative-detection collapses (Fig. 4). The average number of photons in the QMM (Fig. 4(a)) drops where the resonant high-speed peaks appear in Fig. 3(a). The photon number distribution (panels (b1) and (c1) shows the same two-peak structure due to Zeno effect as the speed distribution functions of Fig. 4(c,d), which disappears as the emission rate drops.

## Conclusions

We have proposed a new implementation of Renninger’s negative-result Gedankenexperiment, based on the propagation of photons through a quantum metamaterial (a macroscopic artificial quantum coherent medium). Our simple model simulation demonstrated that the interplay of quantum state collapses of the system due to positive- and negative-result measurements of photons at the exit of the QMM, imposed on top of quantum beats of the QMM between the states with different refractive indices, produces characteristic effects (quantum Zeno-fixing the QMM in the high-*n* (“slow”) state and “anti-Zeno”-fixing it in the low-*n* (“fast”) state, dependent on the ratio between the photon emission rate and quantum beat frequency of the QMM. We suggest that an experimental realization of this scheme would provide a clear visualization of quantum state collapse produced by a “non-measurement”. While not contradicting any current interpretation of quantum mechanics, we believe that such behaviour is more naturally described in terms of a “real”, nonlocal quantum state of the system “quantum metamaterial + photons”.

## References

[1] Namiki, M., Pascazio, S. & Nakazato, H. Decoherence and Quantum Measurements (World Scientific, 1997).

[2] Jammer, M. The Philosophy of Quantum Mechanics: The Interpretations of Quantum Mechanics in Historical Perspective. (Wiley, 1974).

[3] Clerk A, Devoret M, Girvin S, Marquardt F, Schoelkopf R (2010). Rev. Mod. Phys..

[4] Mooij JE (1999). Science.

[5] Braginsky, V. & Khalili, F., Quantum Measurement (Cambridge University Press, Cambridge, 1992).

[6] Zagoskin, A. Quantum Engineering: Theory and Design of Quantum Coherent Structures (Cambridge University Press, 2011).

[7] Ollivier H, Poulin D, Zurek W (2005). Phys. Rev. A.

[8] Renninger M (1953). Zeitschrift fuer Physik.

[9] De Baere, W. On wave-particle duality (english translation of renninger’s article) https://arxiv.org/abs/physics/0504043 (2005).

[10] Cramer J (1986). Rev. Mod. Phys..

[11] Dicke R (1981). Am. J. Phys..

[12] Zagoskin AM (2013). Scientific Reports.

[13] Pusey M, Barrett J, Rudolph T (2012). Nature Phys..

[14] Colbeck R, Renner R (2012). Phys. Rev. Lett..

[15] Hardy L (2013). Int. J. Mod. Phys. B.

[16] Patra M, Pironir S, Massar S (2013). Phys. Rev. Lett..

[17] Ringbauer M (2015). Nature Phys..

[18] Rakhmanov, A. L., Zagoskin, A. M., Savel’ev, S. & Nori, F. *Phys. Rev*. B **77** ISSN 1098–0121 (2008).

[19] Macha, P. *et al*. *Nature Communications* **5**, 5146 (2014). 10.1038/ncomms6146 25312205

[20] Kakuyanagi, K. *et al*. *Phys. Rev. Lett*. **117**, 210503, 10.1103/PhysRevLett.117.210503 (2016). 10.1103/PhysRevLett.117.210503 27911564

[21] Sudarshan ECG, Misra B (1977). J. of Math. Phys..

